# A Vasoactive Role for Endogenous Relaxin in Mesenteric Arteries of Male Mice

**DOI:** 10.1371/journal.pone.0107382

**Published:** 2014-09-22

**Authors:** Chen Huei Leo, Maria Jelinic, Jon H. Gooi, Marianne Tare, Laura J. Parry

**Affiliations:** 1 Department of Zoology, The University of Melbourne, Parkville, VIC, Australia; 2 Department of Physiology, The University of Melbourne, Parkville, VIC, Australia; 3 Department of Physiology, Monash University, Clayton, VIC, Australia; University of Padua, Italy

## Abstract

The peptide hormone relaxin has striking effects on the vascular system. Specifically, endogenous relaxin treatment reduces myogenic reactivity through nitric oxide (NO)-mediated vasorelaxation and increases arterial compliance in small resistance arteries. However, less is known about the vascular roles of endogenous relaxin, particularly in males. Therefore, we used male wild-type (*Rln* ^+/+^) and relaxin knockout (*Rln* ^−/−^) mice to test the hypothesis that passive wall properties and vascular reactivity in mesenteric arteries would be compromised in *Rln* ^−/−^ mice. Passive compliance was determined in arteries (n = 8–9) mounted on a pressure myograph and in Ca^2+^-free Krebs containing 2 mM EGTA. Passive volume compliance was significantly (*P* = 0.01) decreased in the mesenteric arteries of *Rln* ^−/−^ mice. Vascular reactivity was assessed using wire myography. In mesenteric arteries (n = 5) of *Rln* ^−/−^ mice, there was a significant (*P*<0.03) increase in sensitivity to the vasoconstrictors phenylephrine and thromboxane-mimetic U41669. This enhanced responsiveness to vasoconstrictors was abolished by endothelial denudation, and attributed to impaired NO and prostanoid pathways in *Rln* ^−/−^ mice. Sensitivity to the endothelial agonist acetylcholine was significantly (n = 7–9, *P*≤0.03) decreased, and this was abolished in the presence of the cyclooxygenase inhibitor, indomethacin (2 µM). This indicates that prostanoid vasoconstrictor pathways were upregulated in the mesenteric arteries of *Rln* ^−/−^ mice. In summary, we demonstrate endothelial dysfunction and impaired arterial wall remodeling in male mice deficient in relaxin. Thus, our results highlight a role for endogenous relaxin in the maintenance of normal mesenteric artery structure and function in males.

## Introduction

The 6 kDa peptide hormone relaxin is considered a pregnancy hormone because this is when highest circulating concentrations of relaxin are measured. It is also involved in some of the key maternal adaptations to pregnancy [1]. Some of these beneficial effects of relaxin are associated with hemodynamic changes in the renal and systemic vasculature [2]. These include increased glomerular filtration rate, global arterial compliance and cardiac output, and reduced systemic vascular resistance [3], [4], and are attributed to the vasodilatory effects of relaxin on small resistance arteries [2]. A vasoactive role for relaxin is supported by our recent data that localized the relaxin receptor, RXFP1 in both endothelial and smooth muscle cells in a variety of rat arteries and veins [5].

Chronic *in vivo* relaxin treatment in normotensive male and non-pregnant female rats reduces myogenic reactivity in small renal and mesenteric arteries [6] and increases flow-mediated vasodilation [7]–[9] through mechanisms involving endothelium-dependent, nitric oxide (NO) [6], [10]. Furthermore, bradykinin (BK)-mediated, endothelium-dependent relaxation is enhanced in the mesenteric arteries of relaxin-treated rats, due to an increase in the contribution of NO [5]. In small resistance arteries, vascular tone is modulated by several endothelium-derived factors, including NO, prostacyclin (PGI_2_) and endothelium-derived hyperpolarization (EDH) [11]–[13]. Relaxin also has rapid actions, with a bolus IV injection (13.33 mg/kg) increasing BK-mediated vasodilation 3 hours later via a mechanism involving upregulation of EDH. Interestingly, the improved BK-mediated vasodilation was also observed 24 hours after bolus injection, with the prolonged response due to increased PGI_2_ contribution [14]. The effects of relaxin treatment on arterial compliance are also well-established. Specifically, chronic relaxin treatment in normotensive rodents increases passive compliance in the small renal and mesenteric arteries [5], [15], [16] and improves carotid artery distensibility in senescent spontaneously hypertensive rats [17]. In brain parenchymal and small renal arteries relaxin causes outward geometric [18] and hypertrophic remodeling [19], respectively. However, very little work has investigated the vascular functions of endogenous relaxin.

In pregnant rats, treatment with a monoclonal antibody-against relaxin (MCA1) neutralizes high levels of circulating relaxin. This prevents many of the renal and hemodynamic changes that occur throughout pregnancy [3]. In particular, stroke volume, cardiac output, global arterial compliance and glomerular filtration rate are not increased in pregnant MCA1-treated rats and systemic vascular resistance is not decreased [3], [20]. Furthermore, MCA1 treatment results in increased uterine artery stiffness [21] and myogenic reactivity in the small renal arteries of late pregnant females [20]. Aged pregnant relaxin gene knockout (*Rln* ^−/−^) mice also have stiffer uterine arteries [22]. These studies illustrate the detrimental effects on the vasculature of a lack of endogenous relaxin during pregnancy. However, relaxin is also locally produced in arteries of non-pregnant female and male rodents, and the small renal arteries of *Rln* ^−/−^ mice have increased myogenic reactivity and reduced passive compliance [23].

The mesenteric artery is a key target of exogenous relaxin action [5], [7], , but no studies to date have assessed the role of endogenous relaxin in this vascular bed. Therefore, we tested the hypothesis that vascular reactivity and passive mechanical wall properties in mesenteric arteries will be compromised in male *Rln* ^−/−^ mice.

## Materials and Methods

### Ethics statement

All animal experiments were approved by The Faculty of Science, University of Melbourne Animal Experimental Ethics Committee (AEC #0911478.1) and conducted in accordance with the Australian Code of Practice and the National Health and Medical Research Council. All efforts were made to minimize animal pain and suffering.

### Animal model

This study used the original *Rln* ^−/−^ mouse [22], [24] backcrossed on a C57/BLK6J background to the F_14_ generation and wild-type littermates (*Rln* ^+/+^) of the same strain. Genotypes were confirmed by PCR analysis of genomic DNA from ear clips as previously described [24]. Mice were housed in the Department of Zoology Animal House Facilities (University of Melbourne) in a 12 h light and dark cycle at 20°C, with standard food pellets (Barastock, VIC, Australia) and water provided *ad libitum*.

### Isolation of mesenteric arteries

Male adult mice (*Rln* ^+/+^ aged 12.63±0.09 months n = 22; *Rln* ^−/−^ mice aged 12.51±0.09 months, n = 18) were euthanized by isofluorane overdose and cervical dislocation. Additional experiments to investigate mechanisms of vascular dysfunction used mice aged 5 months (*Rln* ^+/+^ aged 5.65±0.17 months, n = 9; *Rln* ^−/−^ mice aged 5.00±0.28 months, n = 8). Cardiovascular phenotypes have been shown in *Rln* ^−/−^ mice of these ages [22], [25]. The mesenteric arcade was isolated and immediately placed in ice cold 0.1 M phosphate buffered saline solution (PBS). Several arteries were snap frozen in liquid nitrogen and stored at −80°C for further analysis. Small mesenteric arteries (first-order branch of the superior mesenteric artery, diameter ∼140 µm) were isolated, cleared of fat and loose connective tissue.

### Pressure myography

Mesenteric arteries from *Rln* ^+/+^ (n = 9) and *Rln* ^−/−^ (n = 9) mice were transferred to a Ca^2+^-free physiological saline solution (PSS; mmol/l: NaCl 149, KCl 4.7, NaHCO_3_ 1.7, KH_2_PO_4_ 1.2, MgSO_4_ 1.7, glucose 5, HEPES 10 and EGTA 2). Leak-free segments of vessels were mounted on a pressure myograph (Living Systems Instrumentation, Burlington, VT, USA) and incubated in Ca^2+^-free PSS at 37°C for 20 minutes before wall parameters (vessel length, outer diameter (OD), inner diameter (ID) and wall thickness (WT)), and wall stress and strain were acquired and calculated [26]. For normalization of ID and OD, values were expressed as: (value at pressure –value at 5 mmHg)/(value at 5 mmHg). Volume compliance was calculated for each pressure increment using the following calculation: volume compliance  =  (Δ volume)/(Δ pressure), where Δ volume  =  (Δ cross sectional area) × (Δ length), and cross sectional area  =  (π. ID^2^)/4 [5]. The initial length and volume of the mounted vessel segments were comparable between groups.

### Wire myography

Mesenteric arteries were cut into rings 2 mm in length and mounted on a Mulvany-Halpern wire-myograph (model 610 M, Danish Myo Technology, Aarhus, Denmark). All experiments were performed in Krebs bicarbonate solution (mmol/l: NaCl 120, KCl 5, MgSO_4_ 1.2, KH_2_PO_4_ 1.2, NaHCO_3_ 25, D-glucose 11.1 and CaCl_2_ 2.5) at 37°C and the baths were bubbled with carbogen (95% O_2_ and 5% CO_2_). Testing of vascular reactivity was performed as previously described [5], [14], [27] with the following modifications. Briefly, mesenteric arteries were contracted with high potassium PSS (K^+^ = 100 mmol/l, isosmotic replacement of Na^+^ with K^+^) and the integrity of the endothelium was determined. To evaluate the vascular smooth muscle reactivity to vasoconstrictors, cumulative concentration-response curves to phenylephrine (PE, 1 nmol/l-10 µmol/l) and the thromboxane mimetic U46619 (0.1 nmol/l-1 µmol/l) were constructed. The influence of the endothelium on vascular smooth muscle reactivity to vasoconstrictors was evaluated in endothelium-denuded arteries. The endothelium was considered to be denuded if the response to ACh (10 µmol/l) was diminished by >90%. Similarly, to assess endothelial and vascular smooth muscle vasodilator function, mesenteric arteries were pre-constricted to a similar level (70–80% KPSS) using PE (0.1–3 µmol/l) and cumulative concentration-response curves to the endothelium-dependent agonist ACh (0.1 nmol/l-10 µmol/l) and to the endothelium-independent, NO donor, SNP (0.1 nmol/l-10 µmol/l) were determined. In addition, responses to ACh, PE and U46619 were examined after 20 minutes incubation with different combinations of Nω-nitro-l-arginine methyl ester (L-NAME; 100 µmol/l), a nitric oxide synthase (NOS) inhibitor, indomethacin (Indo; 2 µmol/l), a cyclooxygenase inhibitor, 1-[(2-chlorophenyl)(diphenyl)methyl]-1H-pyrazole (TRAM-34; 1 µmol/l), a selective blocker of the intermediate-conductance calcium-activated K^+^ channel (IK_Ca_) and apamin (1 µmol/l), a small-conductance calcium-activated K^+^ channel (SK_Ca_) inhibitor. Responses remaining in the presence of Indo and L-NAME were attributed to EDH [28]. In a separate set of experiments, basal NOS activity was examined through the addition of L-NAME (200 µmol/l) in endothelium-intact rings submaximally contracted with PE (10 to 100 nmol/l) to 20% of KPSS contraction.

### Quantitative PCR

Frozen blood vessels were placed in pre-chilled Wig-L-Bug capsules with a silver ball bearing and pulverized in a Digital Wig-L-Bug amalgamator (Dentsply-Rinn, Elgin, IL, USA). Pulverized tissues were resuspended in 1 ml TriReagent (Ambion Inc., Scoresbury, VIC, Australia) and total RNA was then extracted [14], [21]. RNA pellets were resuspended in 11 µl RNA Secure (Ambion). Quality and quantity of RNA was analyzed using the NanoDrop ND1000 Spectrophotometer (Thermo Fischer Scientific Australia Pty Ltd, Scoresby, VIC, Australia) with A_260_:A_280_ ratios >1.8 indicating sufficient quality for qPCR analysis. First strand cDNA synthesis used 0.5 µg of total RNA in a 20 µl reaction containing random hexamers (50 ng/µl) and 200 units of Superscript III (Invitrogen, Mulgrave, VIC, Australia).

The comparative cycle threshold (2^−ΔCt^) method of quantitative real-time polymerase chain reaction (qPCR) was used to further explore the mechanisms underlying the up-regulation of vasoconstrictor prostanoids. Cyclooxygenase 1 (*Ptgs1*), and 2 (*Ptgs2*) and thromboxane synthase (*Tbxas1*), and receptor (*Tbxa2r*) gene expression in *Rln* ^+/+^ and *Rln* ^−/−^ mice was analyzed (Table 1). Mouse-specific forward/reverse primers and 6-carboxyl fluorescein–labelled (FAM) Taqman probes were designed and purchased from Biosearch Technologies (Novato, CA, USA). Primers were designed to span intron/exon boundaries and avoid *Rxfp1*-truncates. qPCR was performed on the the AB Applied Biosystems ViiA7 PCR machine (Life Technologies, Mulgrave, VIC, Australia) using 96-well reaction plates with 20 µl volume reactions in triplicate containing SensiFast Probe Lo-ROX master mix (Bioline) and 10 µM of primers and FAM-labelled probe. Ribosomal 18S (*Rn18s*) was the reference gene. Negative template controls substituting cDNA with water or RT negative controls substituting the reverse transcriptase in the cDNA synthesis, were included on each plate. For each sample, the mean *Rn18s* C_T_ triplicate value was subtracted from the mean gene of interest triplicate C_T_ value to normalize gene of interest expression to the reference gene. These normalized data (ΔC_T_) were then presented as a relative value (mean ± SEM).

**Table 1:** Primer and probe sequences for quantitative real time PCR experiments.

Gene/Accession ID		Sequence 5′ to 3′	Position	Amplicon Length
*Rn18s* NM	Fwd	GCATGGCCGTTCTTAGTTGG	1330	77 bp
	Rev	TGCCAGAGTCTCGTTCGTTA	1377	
	Probe	TGGAGCGATTTGTCTGGTTATTCCGA	1350	
*Ptgs1* NM_008969.3	Fwd	TCGGTCCTGCTCGCAGAT	204	95 bp
	Rev	AGGCCAAAGCGGACACAGA	280	
	Probe	CACCAGTCAATCCCTGTTGTTA	235	
*Ptgs2* NM_011198.3	Fwd	TCCTCCCGTAGCAGATGAC	514	158 bp
	Rev	TGCTGGGCAAAGAATGCAAAC	649	
	Probe	GGGAAATAAGGAGCTTCCTGAT	556	
*Tbxa2r* NM_009325.4	Fwd	GACTGCGAGGTGGAGATGAT	1159	116 bp
	Rev	ATGACAGGTGGTGTCTGCAA	1274	
	Probe	CCCTTGCTGGTCTTCATCAT	1225	

### Vascular reactivity reagents

All drugs were purchased from Sigma-Aldrich (St Louis, MO, USA), except for U46619 (Cayman Chemical, Ann Arbor, MI, USA). They were all dissolved in distilled water, with the exception of indomethacin, which was dissolved in 0.1 mol/l sodium carbonate, TRAM-34, which was dissolved in 100% DMSO (final concentration less than 0.1% DMSO), and U46619, which was dissolved in 100% ethanol (final concentration less than 0.1% ethanol) as 1 mmol/l stock solution and subsequent dilutions were in distilled water.

### Statistical analysis

All results are expressed as the mean ± SEM, n represents the number of animals per group. Concentration response curves from mouse mesenteric arteries were fit to a sigmoidal curve using nonlinear regression (Prism version 5.0, GraphPad Software, San Diego, CA, USA) to calculate the sensitivity of each agonist (pD_2_). Maximum relaxation (R_max_) to vasodilators was measured as a percentage of pre-constriction to PE. Cohort pD_2_ and R_max_ values were compared via one-way ANOVA with post-hoc analysis using Dunnett's test or Student's independent t-test as appropriate. The stress-strain curves and pressurized wall parameters (WT, OD, ID) were analyzed with repeated measures two-way ANOVA (treatment vs. strain). Volume compliance was analyzed using a two-way ANOVA, with Bonferroni post-hoc analysis. A level of *P*<0.05 was considered statistically significant.

## Results

### Arteries of *Rln* ^−/−^ mice have reduced volume compliance

**Figure 1:**
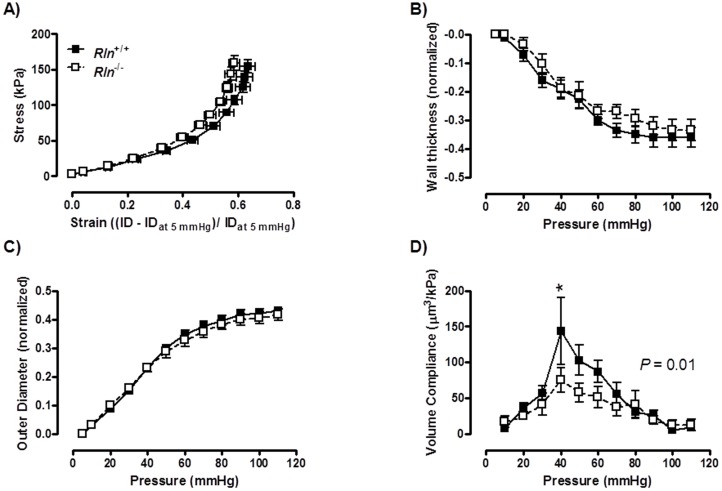
Relaxin deficiency reduces volume compliance. Stress - strain relationships (A), and normalized passive wall thickness (WT; B), outer diameter (OD; C) and volume compliance (D) against intraluminal pressures in mesenteric arteries from *Rln^+/+^* (▪) and *Rln* ^−/−^ (□) mice. Values are mean ± SEM. * significantly (*P* = 0.01) less than *Rln^+/+^* mice, *n* = 8–9.

There was no significant difference between genotypes in WT, OD and ID in the mesenteric artery at baseline (5 mmHg; Table 2). Furthermore, over the pressurization range there was no significant difference in any of the wall parameters (WT, OD and ID) or the stress-strain relationship in the mesenteric artery, indicating that circumferential wall stiffness was comparable between groups (Figure 1A–C). Conversely, volume compliance was significantly (F_1, 10_ = 5.604; *P* = 0.01) reduced in mesenteric arteries of *Rln* ^−/−^ mice compared with *Rln^+/+^* (Figure 1D).

**Table 2:** Age, bodyweight, and mesenteric artery dimensions and vascular reactivity for 12 month old wild-type (*Rln* ^+/+^) and relaxin-deficient (*Rln* ^−/−^) mice.

	*Rln* ^+/+^	N	*Rln* ^−/−^	N
**Age (months)**	12.63±0.09	22	12.51±0.09	18
**Body weight (g)**	35.99±0.74	19	35.15±0.83	17
**Stress- Strain K-value**	5.04±0.26	8	5.40±0.32	6
**Baseline OD (µm)**	143.2±5.3	9	142.1±9.3	9
**Baseline ID (µm)**	114.5±5.1	9	116.0±7.9	9
**Baseline WT (µm)**	14.3±0.8	9	13.1±0.9	9
**Baseline length (µm)**	929.5±104.1	9	812.7±111.8	9
**Baseline volume (µm^3^)**	921.9±85.5	8	229.2±223.1	9
**Hi K E_max_** **(mN/mm)**	8.96±0.38	14	8.57±0.56	14
**PE E_max_** **(% Hi K)**	112.4±3.9	9	124.5±2.9*	10
**PE E_max_** **(denuded) (% Hi K)**	121.8±6.5	7	131.5±7.6	6
**U46619 E_max_** **(% Hi K)**	125.4±4.4	8	132.3±4.9	5
**U46619 E_max_** **(denuded) (% Hi K)**	132.8±7.1	5	137.3±8.1	4
**ACh R_max_** **(%)**	85.54±3.75	8	92.56±2.13	7
**ACh (+ Indo) R_max_ (%)**	77.07±4.03	8	89.75±4.25	6
**ACh (+ L-NAME & Indo) pD_2_**	6.87±0.28	6	6.26±0.13	5
**ACh (+ L-NAME & Indo) R_max_ (%)**	45.99±11.49	6	49.38±5.72	6
**ACh (+Indo & T34&Apa) pD_2_**	6.77±0.26	5	6.40±0.26	5
**ACh (+ Indo & T34&Apa) R_max_ (%)**	38.21±4.46	5	42.45±10.72	5
**ACh (+Indo, L-NAME& T34&Apa) pD_2_**	N/D	5	N/D	6
**ACh (+Indo, L-NAME & T34&Apa) R_max_ (%)**	6.91±6.91	5	3.70±1.78	6
**SNP R_max_ (%)**	99.70±0.67	9	101.10±0.75	7
**L-NAME induced contraction**	35.47±6.67	7	30.81±4.51	5

Baseline (at 5 mmHg) artery dimensions: outer diameter (OD), inner diameter (ID) and wall thickness (WT). Vascular reactivity data: phenylephrine (PE); acetylcholine (ACh); 100 mM potassium PSS (Hi K); nitric oxide synthase inhibitor (L-NAME); indomethacin (Indo); 1-[(2-chlorophenyl) (diphenyl) methyl]-1H-pyrazole and apamin (T34 & Apa); maximum response (E_max_); maximum relaxation (R_max_); pD2 sensitivity (−log EC50); N/D not determined. All values are expressed as mean ± SEM and N refers to the sample size. * significantly (*P*<0.05) greater than *Rln* ^+/+^ mice.

### Arteries of *Rln* ^−/−^ mice are more responsive to PE and U46619

**Figure 2:**
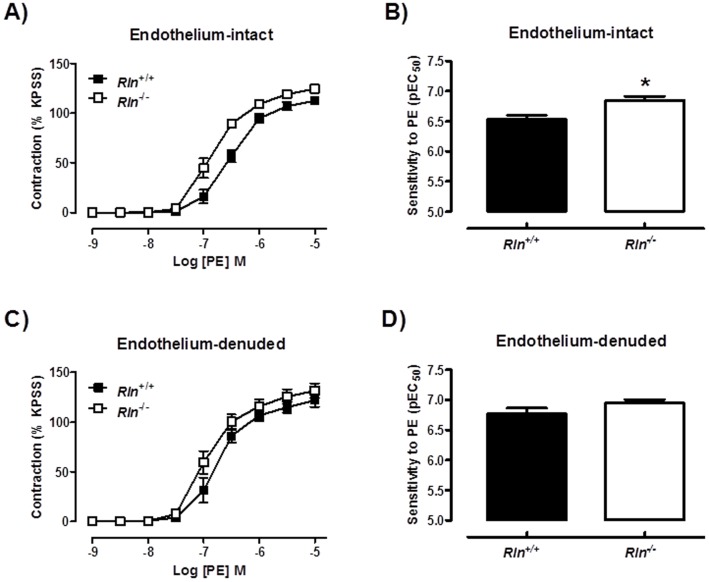
Relaxin deficiency increases PE-mediated contraction. Concentration-response curves and sensitivity (pEC_50_) to phenylephrine (PE) in endothelium-intact (A & B) and endothelium-denuded (C & D) mesenteric arteries from 12 month old *Rln^+/+^* (▪) and *Rln* ^−/−^ (□) mice. Values are mean ± SEM. * significantly (*P* = 0.006) greater than *Rln^+/+^* mice, *n* = 6–10.

**Figure 3:**
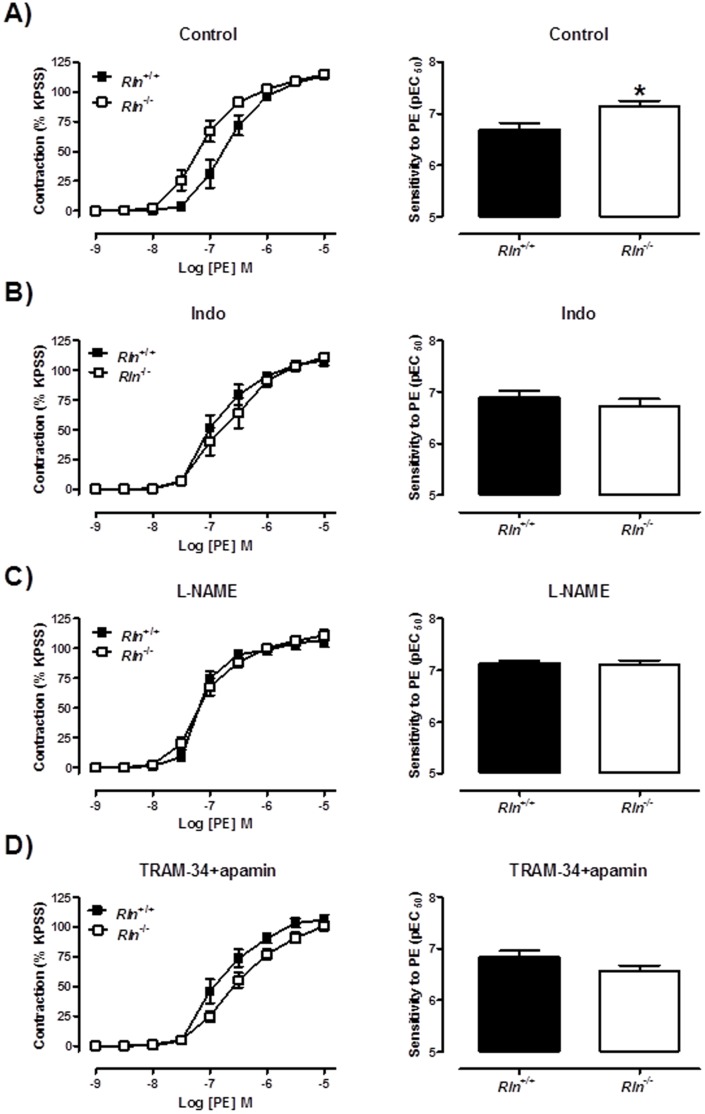
Mechanisms of enhanced PE-mediated contraction in relaxin deficient mice. Concentration-response curves and sensitivity (pEC_50_) to phenylephrine (PE) in endothelium-intact mesenteric arteries from 5 month old *Rln^+/+^* (▪) and *Rln* ^−/−^ (□) mice. Responses were assessed in the absence of inhibitors (A), and presence of indomethacin (B), L-NAME (C) or TRAM-34 and apamin (D). Values are mean ± SEM. * significantly (*P* = 0.01) greater than *Rln^+/+^* mice, *n* = 8.

Sensitivity (pD_2_) to PE in endothelium-intact mesenteric arteries was significantly (t_17_ = 3.17, *P* = 0.006) increased in *Rln* ^−/−^ mice compared with *Rln* ^+/+^ mice (Figure 2A & B). Maximum contraction was also increased (t_17_ = 2.54, *P* = 0.02) in arteries of *Rln* ^−/−^ mice compared with *Rln* ^+/+^ mice (Table 2). These differences were no longer significant when the endothelium was removed (Figure 2C & D), indicating that the increase in responsiveness to PE in *Rln* ^−/−^ mice was endothelium-dependent. Furthermore, in endothelium-intact mesenteric arteries, the enhanced sensitivity (t_14_ = 2.84, *P* = 0.01) to PE in *Rln* ^−/−^ mice (Figure 3A) was not significantly different in the presence of Indo, (Figure 3B) L-NAME (Figure 3C) or TRAM34 + apamin (Figure 3D).

**Figure 4:**
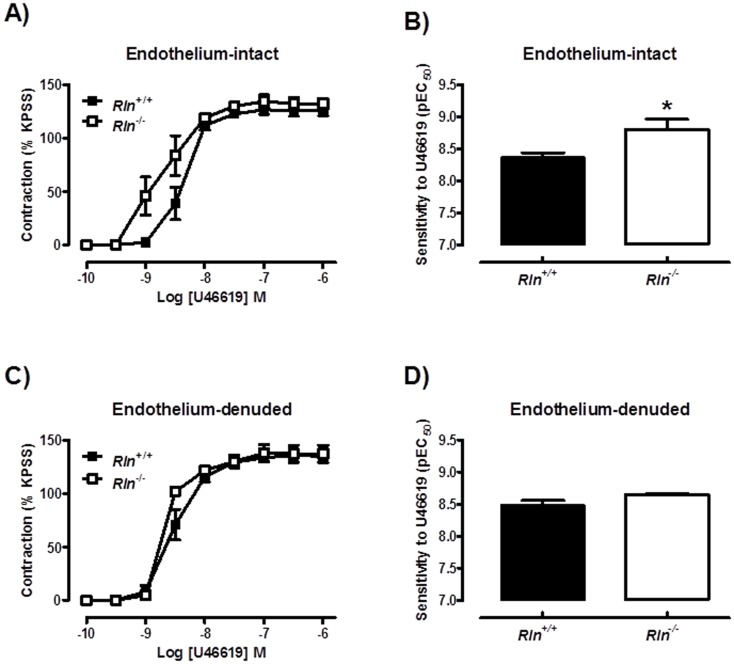
Relaxin deficiency increases U46619-mediated contraction. Concentration-response curves and sensitivity (pEC_50_) to the thromboxane mimetic U46619 in endothelium-intact (A & B) and endothelium-denuded (C & D) mesenteric arteries from 12 month old *Rln^+/+^* (▪) and *Rln* ^−/−^ (□) mice. Values are mean ± SEM. * significantly (*P* = 0.02) greater than *Rln^+/+^* mice, *n* = 4–8.

**Figure 5:**
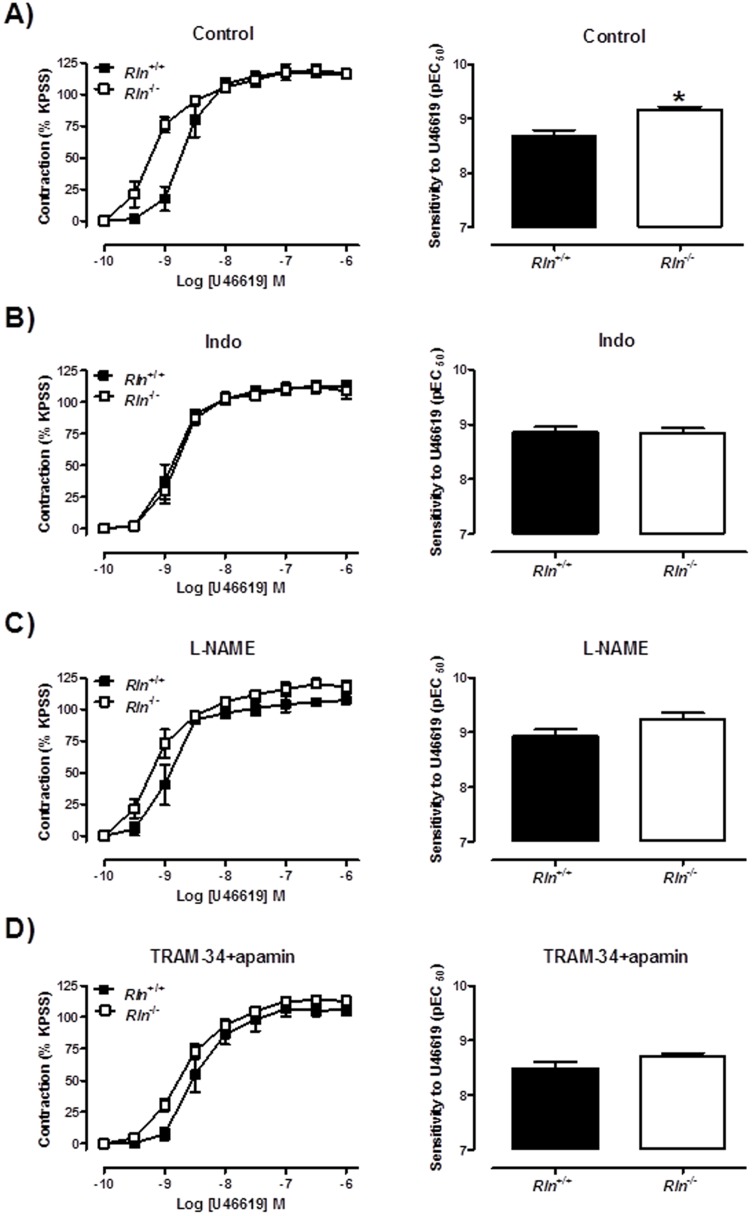
Mechanisms of enhanced U46619-mediated contraction in relaxin deficient mice. Concentration-response curves and sensitivity (pEC_50_) to the thromboxane mimetic U46619 in endothelium-intact mesenteric arteries from 5 month old *Rln^+/+^* (▪) and *Rln* ^−/−^ (□) mice. Parameters were assessed in the absence of inhibitors (A), and presence of indomethacin (B), L-NAME (C) or TRAM-34 and apamin (D). Values are mean ± SEM. * significantly (*P* = 0.004) greater than *Rln^+/+^* mice, *n* = 5–7.

Sensitivity of arteries to U46619 was significantly (t_11_ = 2.68, *P* = 0.02) increased in *Rln* ^−/−^ mice compared with *Rln^+/+^* mice (Figure 4A & B) but maximal contraction to U46619 was comparable between genotypes (Table 2). In endothelium-denuded arteries the increase in U46619 sensitivity between genotypes was not significant indicating that this phenotype is endothelium-dependent (Figure 4C & D). In endothelium-intact mesenteric arteries, the enhanced sensitivity (t_10_ = 3.78, *P* = 0.004) to U46619 in *Rln* ^−/−^ mice (Figure 5A) was not significantly different from *Rln* ^+/+^ mice in the presence of Indo, (Figure 5B) L-NAME (Figure 5C) or TRAM34 + apamin (Figure 5D).

### *Rln* ^−/−^ mouse arteries have endothelial vasodilator dysfunction and enhanced smooth muscle vasodilator function

**Figure 6:**
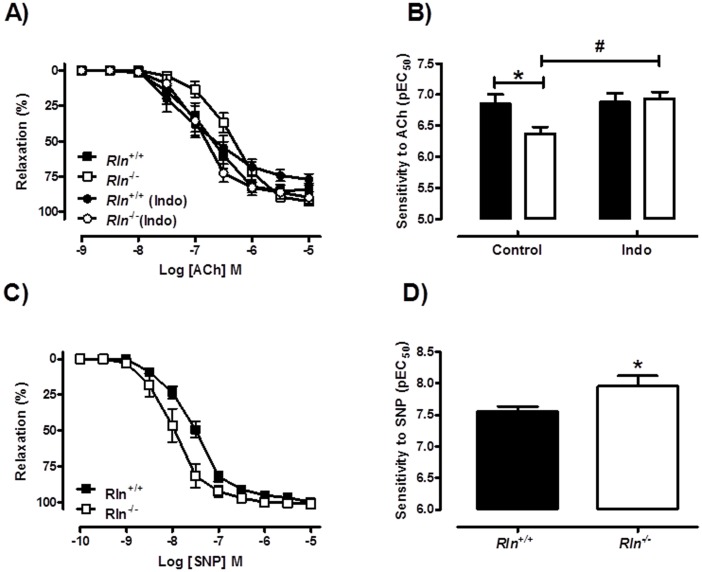
Relaxin deficiency reduces endothelium-dependent relaxation and increases SNP-mediated relaxation. Concentration-response curves and sensitivity (pEC_50_) to acetylcholine (ACh; A & B) and sodium nitroprusside (SNP: C & D) in mesenteric arteries from 12 month old *Rln^+/+^* (▪) and *Rln* ^−/−^ (□) mice. Values are mean ± SEM. * significantly (*P*<0.05) different than *Rln^+/+^* mice, # significantly (P = 0.004) greater than *Rln^+/+^* control, *n* = 6–9.

**Figure 7:**
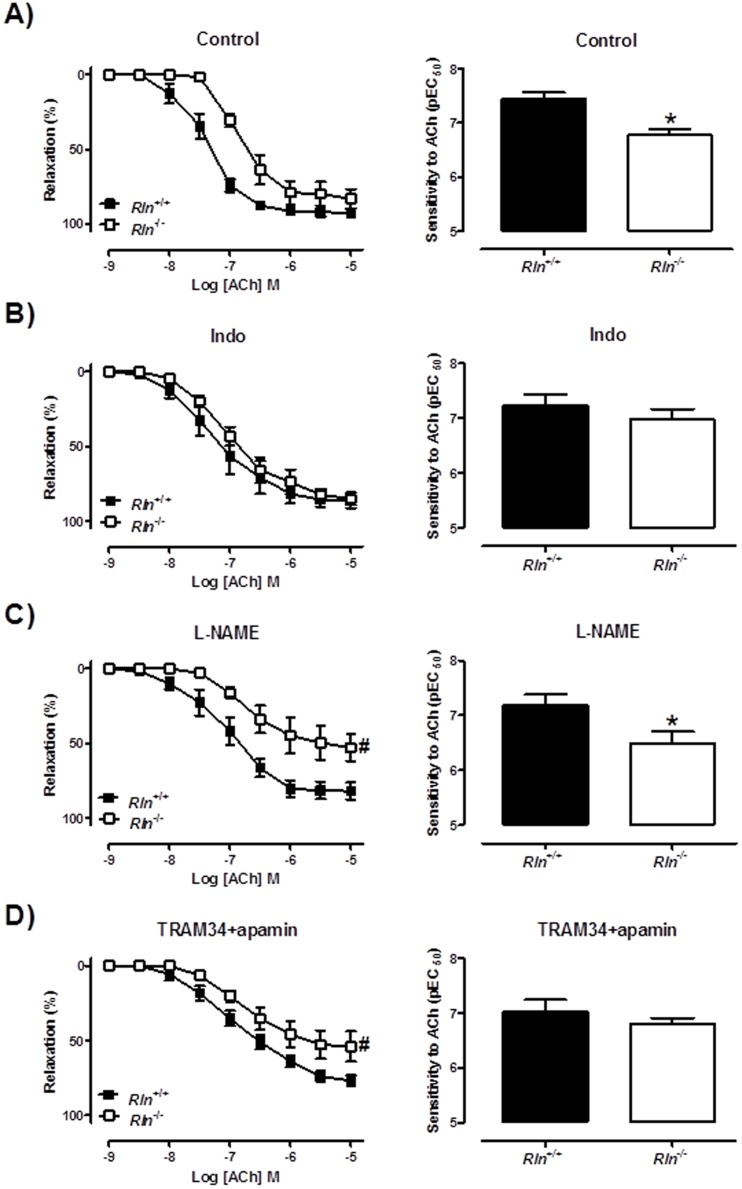
Mechanisms of impaired ACh-mediated contraction in relaxin deficient mice. Concentration-response curves and sensitivity (pEC_50_) to acetylcholine (ACh) in endothelium-intact mesenteric arteries from 5 month old *Rln^+/+^* (▪) and *Rln* ^−/−^ (□) in the absence of inhibitors (A), and presence of indomethacin (B), L-NAME (C) or TRAM-34 and apamin (D).Values are mean ± SEM. # maximum relaxation and * sensitivity significantly (*P*<0.05) less than *Rln^+/+^* mice, *n* = 7–8.

Sensitivity to ACh was significantly (t_13_ = 2.44, *P* = 0.03) decreased in 12 month old *Rln* ^−/−^ mice compared with *Rln* ^+/+^ mice, indicating endothelial dysfunction (Figure 6A & B). Maximum relaxation to ACh was comparable between genotypes (Table 2). The altered sensitivity to ACh in *Rln* ^−/−^ mice was abolished in the presence of the cyclooxygenase inhibitor, Indo (Figure 6A & B). This endothelial dysfunction was also observed in the 5 month old male mice and similarly inhibited by Indo (Figure 7A & B). Furthermore, in the presence of L-NAME, the sensitivity (t_11_ = 2.44, *P* = 0.03) and maximum relaxation (*Rln* ^+/+^: 81.9±5.7% vs. *Rln* ^+/+^: 52.8±9.1%, t_13_ = 2.60, *P* = 0.02) to ACh were still significantly decreased in *Rln* ^−/−^ mice compared with *Rln* ^+/+^ mice (Figure 7C). The L-NAME-induced contraction was comparable between genotypes (Table 2). In the presence of TRAM34 + apamin, the sensitivity (Figure 7D) to ACh was not significantly different in mesenteric arteries from *Rln* ^−/−^ mice. However the maximum relaxation was significantly attenuated (*Rln* ^+/+^: 77±3.4% vs. *Rln* ^+/+^: 54±10%, (t_13_ = 2.28, *P* = 0.04). There were no significant differences between genotypes for ACh-mediated vasodilation in the presence of Indo + L-NAME, Indo + TRAM-34 + apamin and Indo + L-NAME + TRAM-34 + apamin (Table 2). Taken together, these data suggest an upregulation in vasoconstrictor prostanoid pathways in the mesenteric arteries of *Rln* ^−/−^ mice. The NO and EDH components of ACh-mediated relaxation were not significantly affected by relaxin deficiency.

Absolute contraction evoked by high K^+^ physiological saline solution (KPSS, 100 mM) was not affected by genotype at any time point (Table 2). Smooth muscle relaxation to SNP was augmented (Figure 6C). Specifically, sensitivity (Figure 6D) but not maximal relaxation (Table 2) to SNP was significantly (t_13_ = 2.38, *P* = 0.03) increased in mesenteric arteries of *Rln* ^−/−^ mice compared with *Rln* ^+/+^ mice (Table 2).

### *Rln* ^−/−^ mice have unaltered thromboxane receptor and cycloxygenase gene expression

**Figure 8:**
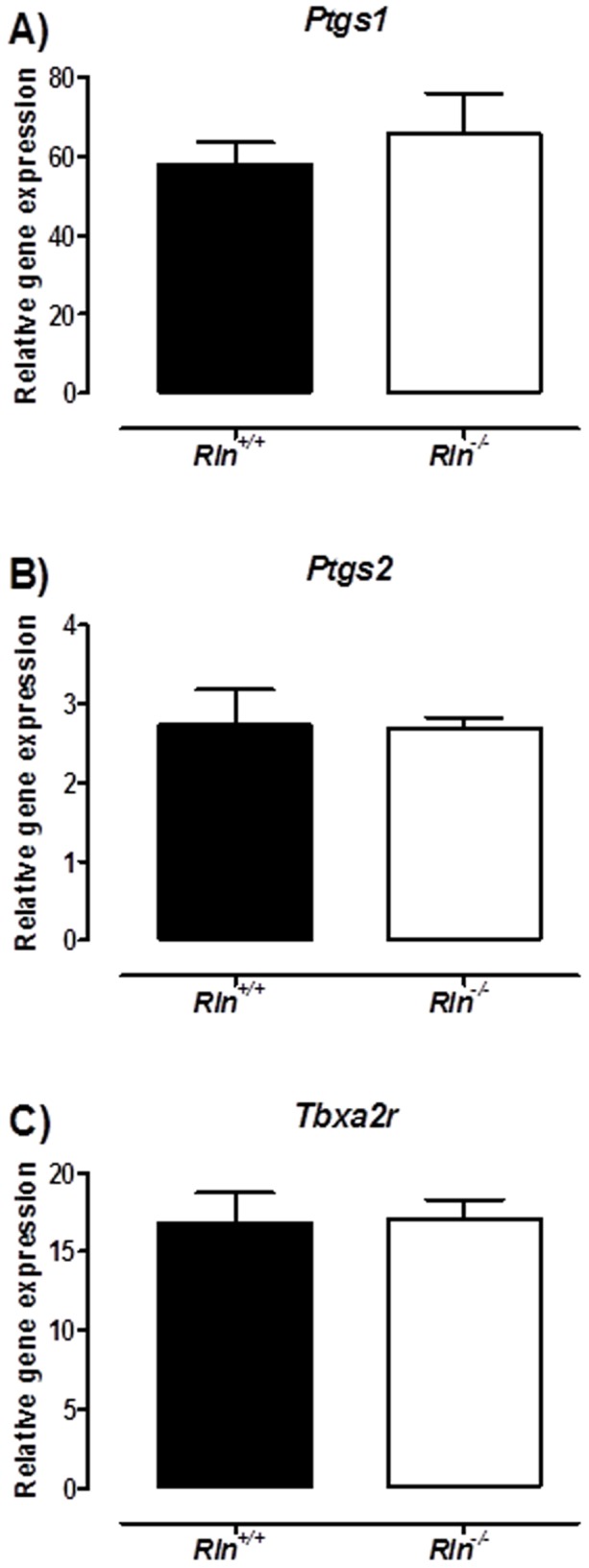
Relaxin deficiency does not affect thromboxane receptor and cyclooxygenase gene expression. Quantitative analysis of cyclooxygenase 1 (*Ptgs1*; A), cyclooxygenase 2 (*Ptgs2*; B) and thromboxane receptor (*Tbxa2r*; C) expression in endothelium-intact mesenteric arteries from *Rln^+/+^* (▪) and *Rln* ^−/−^ (□) mice. Values are mean ± SEM, *n* = 7.

To further explore the mechanisms underlying the upregulation of vasoconstrictor prostanoid pathways in mesenteric arteries of *Rln* ^−/−^ mice, we analyzed gene expression of key regulatory enzymes and receptors. Thromboxane A2 synthase 1 (*Tbxas1*) gene expression was undetectable in mesenteric arteries of either genotype using qPCR. COX1 (*Ptgs1*), COX2 (*Pgts2*) and thromboxane A2 receptor (*Tbxa2r*) gene expression were not significantly different between genotypes (Figure 8).

## Discussion

Our study demonstrates that a lack of endogenous relaxin compromises vessel wall properties and endothelial function in the mesenteric arteries of male mice. Specifically, mesenteric arteries of *Rln* ^−/−^ mice had reduced volume compliance, an endothelium-dependent increase in PE and U46619-evoked contraction and a reduction in endothelium-dependent relaxation. The enhanced responsiveness to vasoconstrictors was associated with impaired NO and prostanoid pathways in *Rln* ^−/−^ mice, whereas impaired ACh-mediated relaxation was attributed to the upregulation of vasoconstrictor prostanoid pathways. Overall this study demonstrates that endogenous relaxin has a vasoactive role in the mesenteric artery of non-pregnant animals.

Similar to previous findings in the small renal artery [19], our data demonstrated that a lack of endogenous relaxin in mice caused a reduction in the volume compliance of mesenteric arteries. Interestingly, stress-strain curves were comparable between genotypes, suggesting no changes in circumferential arterial stiffness. There were no differences in WT, OD or ID, thus geometrical remodeling was comparable between genotypes. Arterial compliance, (a measure of volume change in response to pressure change) is relative to the initial arterial volume [29], and there were no significant differences in initial length or volume between genotypes. Thus our results suggest that endogenous relaxin regulates arterial wall properties that determine arterial lengthening. This could have been due to differences in compositional remodeling or re-arrangement of extracellular matrix fibers involved in arterial lengthening. We previously explored the possibility that relaxin deficiency results in increased collagen in the vessel wall. However, as we showed in the uterine arteries of MCA1-treated pregnant rats [21], there was no evidence of increased collagen in arteries of aged pregnant *Rln* ^−/−^ mice [22]. Instead, they had reduced elastin, matrix metalloproteinase (MMP) expression and pro-MMP-2 activity [22]. The small mesenteric arteries of *Rln* ^−/−^ male mice also have reduced pro-MMP-2 and MMP-9 content [19]. Interestingly, MMP-2 and MMP-9 activity were not altered in these mice, but collagen content was increased. Due to limited tissue availability, it was not possible to analyze MMP activity or collagen and elastin content in the mesenteric arteries in our study. Therefore, the mechanism by which endogenous relaxin mediates vascular remodeling in the mesenteric arteries is yet to be defined.

No studies to date have analyzed vascular reactivity in arteries from *Rln^+/+^* and *Rln* ^−/−^ mice. Our data clearly demonstrate that a lack of endogenous relaxin increased sensitivity to PE and U46619 resulting in enhanced contraction, whereas it only affected maximum response to PE. This enhanced vasoconstrictor responsiveness was dependent on the endothelium and associated with impairment of NO and prostanoid pathways. Similar endothelium-dependent augmentation of α-adrenoceptor vasoconstriction occurs in the aorta of spontaneously hypertensive rats and is thought to be mediated by endothelial COX-2 derived vasoconstrictors including PGF_2α_ and 8-isoprostane [30]. Mesenteric arteries of *Rln* ^−/−^ mice also exhibited endothelial vasodilator dysfunction. In many studies relaxin appears to act through NO signaling [31], [32], so we predicted that the impaired vasorelaxation in *Rln* ^−/−^ mice would be primarily due to a reduction in the NO contribution. However, the reduction in ACh-mediated vasorelaxation was not associated with impaired NO or EDH but rather driven by an upregulation in the actions of vasoconstrictor prostanoid pathways. Endothelial vasodilator dysfunction due to increased production of endothelial COX-derived constrictor factors is a feature of endothelial dysfunction associated with ageing and disease in arteries from animals models and humans [33]. Moreover, recent data in relaxin-treated male rats demonstrate that BK-mediated vasodilation is enhanced through increased PGI_2_-mediated relaxation [14]. To investigate the mechanism underpinning the increased vasoconstrictor prostanoids pathways in *Rln* ^−/−^ mice we analyzed the expression of COX1 (*Ptgs1*), COX2 (*Ptgs2*) and the thromboxane receptor (*Tbxa2r*). Our qPCR data showed no differences between genotypes. It is possible that relaxin stimulates production of vasoconstrictor prostanoids but lack of tissue precluded investigation of the likely candidates, prostaglandin E2 and thromboxanes.

Interestingly, relaxin deficiency also altered vascular smooth muscle vasodilator function. SNP-mediated vasorelaxation was increased in *Rln* ^−/−^ mice and suggests upregulation of the guanylate cyclase-cGMP pathway. This could be due to increased expression or activity of soluble guanylate cyclase or increased sensitivity of the enzyme through redox modulation [34], [35]. In support of our hypothesis, cGMP and NO levels were increased in bovine aortic vascular smooth muscle cells treated with porcine relaxin [36]. We suggest that the upregulation of the smooth muscle guanylate cyclase-cGMP pathway may be a compensatory mechanism to counter the effects of enhanced sensitivity to vasoconstrictors and endothelial vasodilator dysfunction.

The current literature identifies the small renal arteries as a key target for endogenous relaxin [19], [23]. Our data also highlight the mesenteric arteries as a key vascular target for this hormone. These resistance arteries play a critical role in the regulation of haemodynamics, in particular, the control of systemic vascular resistance and blood pressure. Despite evidence of cardiac hypertrophy, *Rln* ^−/−^ mice aged between 8 and 24 months have normal heart rate and blood pressure (mean arterial pressure) [25], though all measurements to date have been made in anaesthetized animals. Interestingly, blocking endogenous relaxin (via MCA1) in conscious pregnant rats reduces stroke volume and cardiac output, but similarly does not alter heart rate or mean arterial pressure [3]. Therefore the impact of endothelial dysfunction in the mesenteric arteries of *Rln* ^−/−^ mice on the cardiovascular system is not clear.

In summary, our study identifies novel vascular phenotypes in the mesenteric arteries of relaxin deficient mice demonstrating that endogenous relaxin is involved in the maintenance of endothelial function and vascular remodeling. The functional consequences on the cardiovascular system are still unclear. However, the lifespan of *Rln* ^−/−^ mice is not shortened as they age (Jelinic, unpublished). We suggest that relaxin deficiency may render individuals vulnerable to, or exacerbate the progression of, cardiovascular disease.
